# Examining determinants of control of metabolic syndrome among older adults with NCDs receiving service at NCD Plus clinics: multilevel analysis

**DOI:** 10.1186/s12913-024-11562-3

**Published:** 2024-09-27

**Authors:** Nongnuch Suapumee, Acharaporn Seeherunwong, Napaporn Wanitkun, Natkamol Chansatitporn

**Affiliations:** 1 Faculty of Nursing, Mahidol University, Bangkok, Thailand; 2 Department of Mental Health and Psychiatric Nursing, Faculty of Nursing, Mahidol University, Bangkok, Thailand; 3 Department of Surgical Nursing, Faculty of Nursing, Mahidol University, Bangkok, Thailand; 4 Department of Biostatistics, Faculty of Public Health, Mahidol University, Bangkok, Thailand

**Keywords:** Metabolic syndrome, Older adults, Non-communicable diseases, Diabetes Mellitus, Hypertension, Hyperlipidemia, Drug adherence, Multilevel analysis, Health service system, NCD clinics, Polypharmacy

## Abstract

**Background:**

Metabolic syndrome (MetS) in older adults with hypertension, diabetes, and hyperlipidemia increases the risks of cardiovascular diseases by 2.5 times and type 2 diabetes by five times. This study aimed to explain the multilevel relationships between health service system factors and individual-level factors influencing the control of MetS among older adults with NCDs receiving health care services at the NCD Plus clinics of hospitals in 1 year.

**Methods:**

This cross-sectional analytical study employed a systematic sampling method to have two groups of samples from 4 regions of Thailand: (1) 600 older adults having at least one diagnosis of NCDs receiving services at NCD Plus clinics and (2) 12 nurses in charge of the NCD Plus clinics at the hospitals providing services to these patient samples. Data were analyzed using multilevel logistic regression analysis.

**Results:**

24% of older adults with NCDs can control MetS within one year. The MetS escalation from the initial assessment to 1-year follow-up varied according to the level of the hospitals. The transition from MetS to non-MetS status was rare in older adults with NCDs. Among health service system factors, complete screening for MetS influenced 1-year MetS control (95% CI [1.06, 2.92]). Older adults who were female and who had polypharmacy had a 66% (95% CI [0.22, 0.53]) and a 54% (95% CI [0.29 − 0.71]) reduction chance in MetS control. Older adults, who were ≥ 80 years old, labor-employed, healthy dietary patterns, and medication adherence increased chances of controlling MetS by 2.38 times (95% CI [1.12, 5.05]), 2.14 times (95% CI [1.03, 4.42]), 1.61 times (95% CI [1.06–2.46]), and 3.18 times (95% CI [1.51, 6.70]), respectively.

**Conclusions:**

NCDs Plus clinics that provide complete screening for MetS significantly enhance their effectiveness in reducing the proportion of older adults with MetS. In addition, the service should pay attention to older adults who are female, are retired, and take multiple medications to achieve MetS control better. The insights gained from such an analysis could be instrumental in pinpointing the resources necessary to bolster the efficacy of NCD Plus clinics.

**Supplementary Information:**

The online version contains supplementary material available at 10.1186/s12913-024-11562-3.

## Background

Metabolic Syndrome (MetS) is becoming a global problem, especially in older adults with non-communicable diseases (NCDs) having a large number of MetS [1]. MetS is a group of abnormal metabolism presenting with central obesity and at least two abnormal clinical symptoms: high blood sugar levels, high blood pressure, high triglycerides, and low HDL cholesterol (High-Density Lipoprotein cholesterol) [2–4]. Nowadays, a small number of older adults living with NCDs can control MetS. Approximately 18% and 41.5% of older adults with high blood pressure and diabetes in the United States and Singapore could control their MetS, respectively [5, 6]. Concerning the MetS situation in Thailand, adults and older adults had MetS 39.1%, an 18% increase from the 2009 survey (21.1%) [7]. Uncontrolled MetS increases the likelihood of heart failure, stroke, and ischemic heart disease. Importantly, it causes a burden of treatment costs, including an increase in hospital admission rates [8].

MetS may be a risk factor attributed to NCDs and vice versa [9, 10]. However, evidence showed that 45.7% of older adults living with NCDs and accessing hospital services had Mets [4]. Thailand has an older adult population of more than 12 million, accounting for 18.94% of the total population in 2020, which is classified as an aging society [11]. Approximately 49.2% of Thai Older adults had hypertension, and 20.1% had diabetes [12]. Thailand has launched a policy to reduce NCDs by establishing an NCD Clinic in 2014. The NCD Clinic provides treatment services to control blood sugar and blood pressure for adults and older adults with diabetes and hypertension for ambulatory. In 2017, NCD Clinic expanded its service provision of health facilities to provide comprehensive care for preventing or reducing complications, including obesity, cardiovascular disease (CVD), and chronic kidney disease (CKD). Then, the name changed to NCD Plus Clinics. Nevertheless, no data shows the results regarding the control of MetS in people who use NCD Plus clinic services.

MetS control in older adults with NCDs depends on multiple determinants, from the individual to the health service system. The research team adopted the Behavioral model of health services use (BM) (Phase 5) [13] as the conceptual framework to identify the factors that potentially facilitate or impede the control of MetS of older adults with NCDs using services at the NCD Plus clinics. The BM emphasizes that access to health services is best accomplished by focusing on contextual and individual determinants [13]. Access refers to getting to the right providers and services at the right time to promote improved health outcomes [14]. Receiving service at NCD Plus clinics for one year constitutes control of MetS as the evaluated health outcome. The health service system and individual factors are determinative of health outcomes.

The contextual determinants refer to the circumstances and environment of health care access [14]. This study’s contextual determinants include the health service system’s structure and care delivery process. The structure of the health service system in this study consisted of the level of hospital, policy, and the quality of the NCD Plus clinics. Policies and resources used to prevent and control MetS in health service delivery correlate with MetS control [15]. The care delivery process in this study consisted of screening on MetS in conjunction with patterns of giving health education, health behavior monitoring, and connecting services to the communities. Implementing these care processes for older adults significantly improved the ability to control MetS [15–18].

**Fig. 1:**
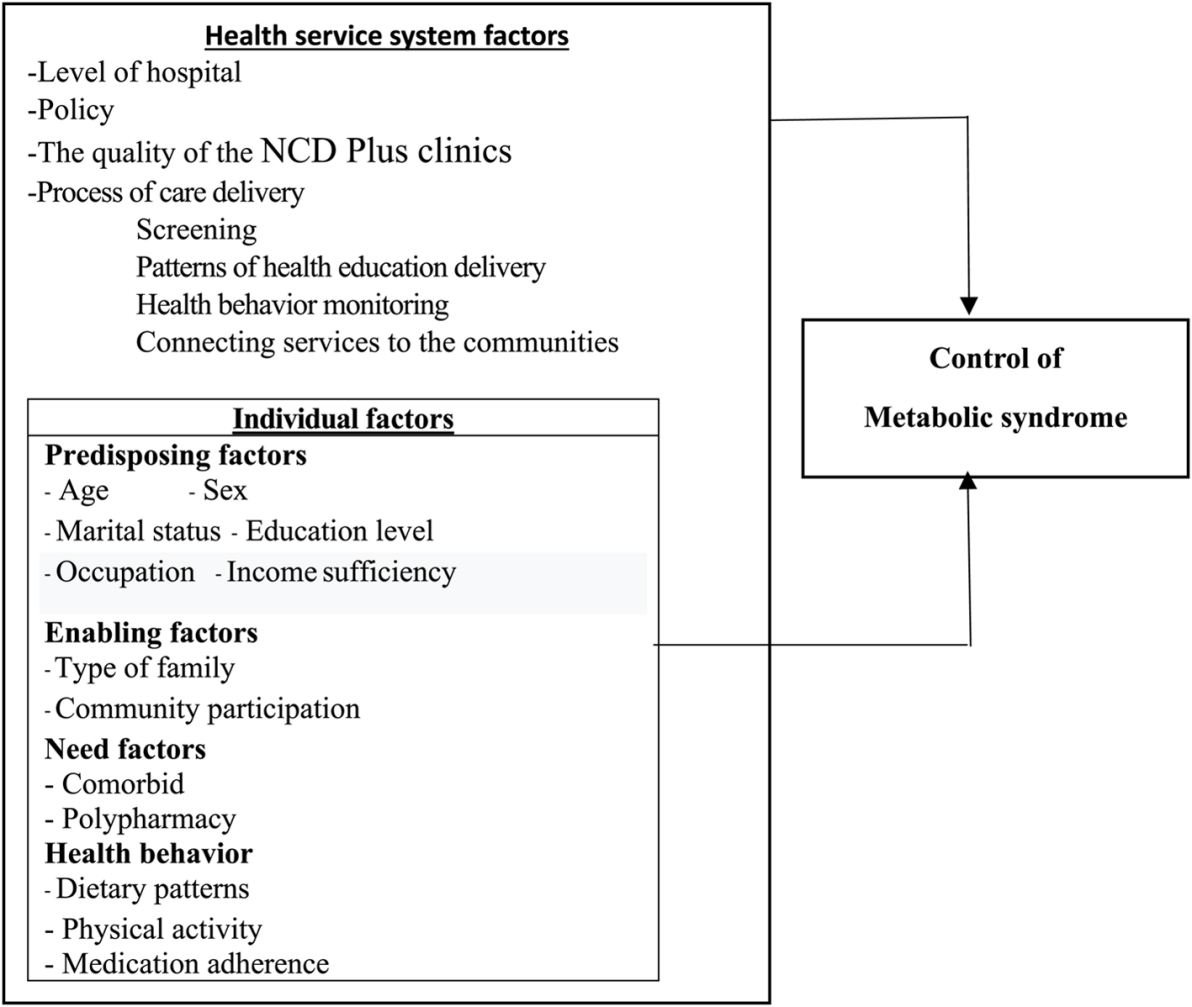
Summarizes the conceptual framework of this study

The contextual determinants can work through individual determinants, improving health outcomes. Individual determinants are a function of predisposing factors, enabling factors, need factors, and health behaviors, as shown in Figure 1. Individual predisposing characteristics, including age, sex, marital status, educational level, occupation, and income sufficiency, can influence MetS control [1, 19]. Enabling factors tend to facilitate or impede control of Mets nested in the service delivery [20], including the family type and perception of community participation in controlling MetS of older adults with NCDs [20, 21]. Family type refers to nuclear families, three-generation families, and skipped-generation households. Need factors were conditions that laypeople or healthcare providers recognized as requiring medical treatment [13]. Comorbidity and polypharmacy are likely to cause less MetS control [22, 23]. Health behaviors, including dietary patterns, physical activities, and medication adherence, were found to increase MetS control among older adults [24, 25].

Diagnosis of MetS in Thailand is based on the diagnostic criteria of the International Diabetes Foundation (IDF) [5]. About ten years ago, 46.8% of older adults had metabolic syndrome [7]. However, there needs to be more information on MetS control in older adults with NCDs in Thailand. Importantly, data reflecting the relationships between individual factors and the health service system influencing MetS still need to be improved. Such data can serve as basic information that is valuable for planning the development of a health service management system responsive to the problems and needs of older adults with NCDs to prevent and control their MetS properly.

Therefore, this study aimed to explore the rate of MetS control in older adults with NCDs and to explain the relationships between the health service system factors and individual factors influencing the control of MetS among older adults with NCDs who received health care services at NCD Plus clinics of the hospitals in 1 year.

## Methods

### Study design and setting

This hospital-based cross-sectional study was conducted from May 25 to June 31/2022, in NCD Plus clinics of 12 public hospitals in four natural regions of Thailand.

### Sampling procedure

**Fig. 2:**
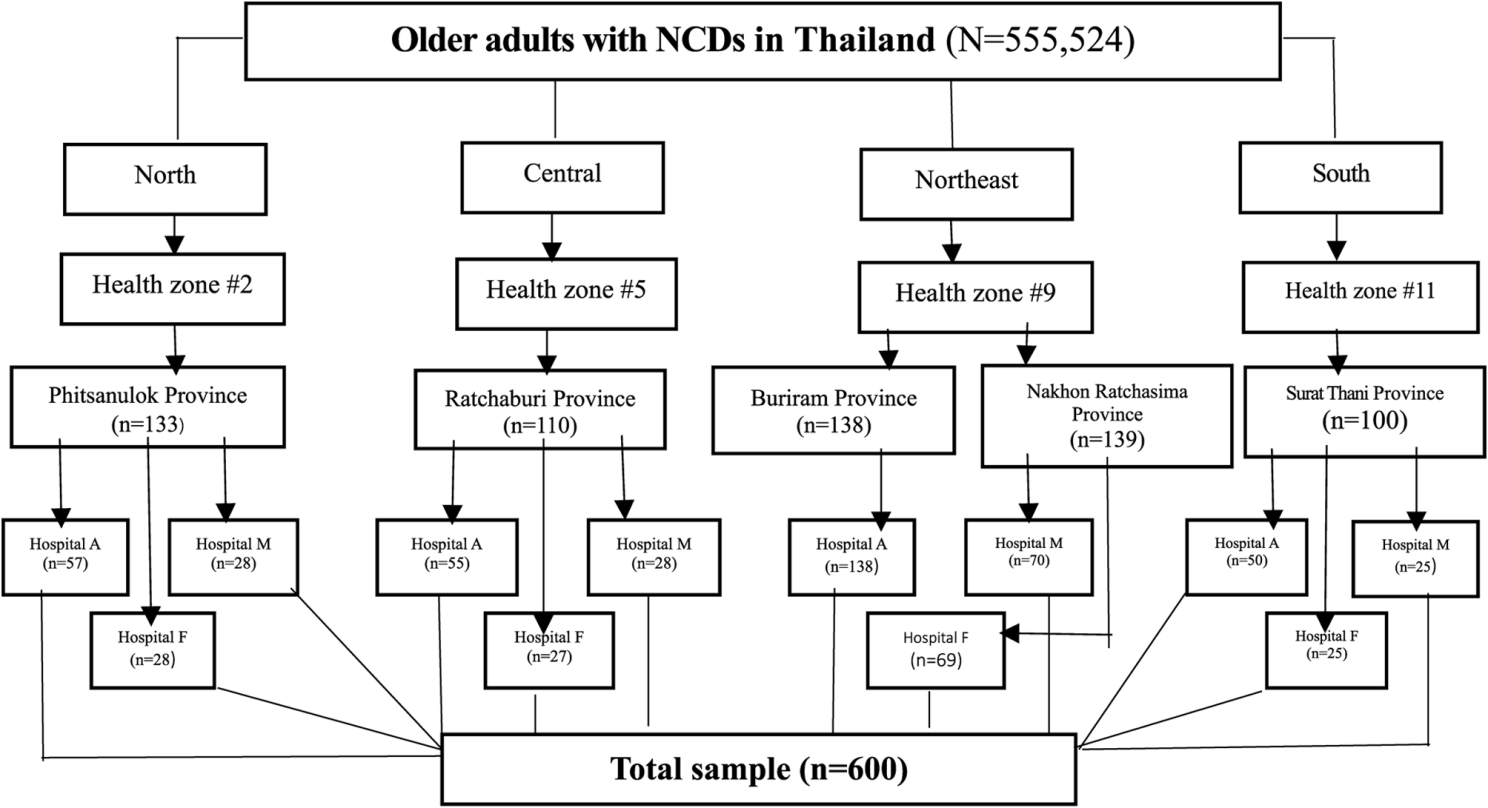
Shows the procedure of random sampling

The unit of analysis in this study was the NCD Plus clinics of hospitals in the Ministry of Public Health. The researchers randomly selected the study areas by applying a multistage sampling method to have the clinics representing the overall picture of NCD Plus Clinics in Thailand, namely the northern, central, northeastern, and southern regions. The researchers chose multistage sampling since it is effective and flexible with a large sample and geographical spread across populations [26]. Thirteen health service areas (Health zones) comprise the health service system of all four regions. Each region has 3–4 health zones. This study required one health zone to represent each region and one province to represent one health zone, in which one health zone covers 5–8 provinces. The sampling process contained the following stages (Fig. 2).

#### Stage 1

Based on the data from the Health Data Center (HDC) database, Ministry of Public Health, researchers selected one health zone in each region with the highest morbidity rate of hypertension and diabetes among older adults over the past three years (2018–2020). As a result, the area can provide the sample size needed to achieve the study’s objectives.

#### Stage 2

Researchers randomly sampled one or two provinces representing each health zone. The province required all-level hospitals, including First–level Hospital (F), Middle-level Hospital (M), and High-level Hospital (A). In Thailand, the Ministry of Public Health classified the hospital level according to their capacity for providing health services in the referral hospital cascade, covering all three levels: F, M, and A-level Hospitals [27]. The researchers randomly selected another province if the random province did not have all 3 level hospitals. Finally, five provinces were obtained.

#### Stage 3

The researchers randomly classified three hospitals, F, M, and A levels, in each province for 12 hospitals. Then, simple random sampling was used to get older adults with NCDs receiving services at NCD Plus clinics at the hospital.

#### Population and samples

The population in this study was older adults aged more than 60 years old who had been diagnosed with at least one disease of hypertension, diabetes, or hyperlipidemia for at least one year and had come to follow up or receive treatments at the NCDs Plus Clinic of hospitals under the Ministry of Public Health. Samples were those who had come to follow up or receive treatments at the NCD Plus clinics of the 12 sampled hospitals, and they had evaluated activities of daily living: ADL ≥ 12 [28].

#### Sample size estimation

The sample size was calculated based on multilevel analysis. The study examined two variables: the health service system and individual characteristic factors affecting the control of MetS among older adults with NCDs. These two-level variables are a group structure in which individual characteristics are embedded in the health service system factors. The sample size must be suitable for the analysis to apply multilevel analysis. In this analysis, the sample size must be at least 20 per study unit [29]. This study determined 12 hospitals as the study areas obtained from multistage sampling. Since the chosen sample size at the individual level was 30 persons per hospital, the subject samples were 12 hospitals X 30 samples = 360 samples.

Multistage sampling requires multiplying the sample size by the Design Effect (DEF) [30]; in this study, DEF was specified = 1.5. Therefore, *n* = 360 × 1.5 = 540.

By using the formula for response error adjustment: n = n/(1-R), the 10% of the sample for response error adjustment was calculated as follows:

The formula substitution: *n* = 540/(1-10) = 60.

Therefore, the sample size used in this study was 540 + 60 = 600 people. All 600 subjects completed data because a researcher collected it herself.

### Variables of interest

#### Control of metabolic syndrome and measurement

Metabolic syndrome was assessed based on conventional cut-off values suggested by the consensus of the IDF diagnostic criteria [31]. According to the diagnosis criteria, older adults with NCDs have MetS when they have abdominal obesity with a waist circumference > 90 cm in males and > 80 cm in females and have at least 2 of 4 following abnormalities: (1) triglycerides ≥ 150 mg/dL or taking medication to lower blood lipid levels, (2) HDL < 40 mg/dL in males and < 50 mg/dL in females or taking medication to lower blood lipid levels, (3) blood pressure ≥ 130/85 mmHg or taking medication to lower blood pressure, and (4) fasting blood glucose ≥ 100 mg /dL or taking medication to lower blood sugar levels. Clinical information was recorded as shown in Supplement I. In this study, the control of MetS was classified into 2 groups: 1 = Yes, 0 = No. The scores were given as ‘1’ if older adults with NCDs never had MetS in the past year or presently had MetS in the past year but are currently transitioning to have no MetS. The scores were given as ‘0’ if the older adults with NCDs had MetS in the past year and still had MetS or never had MetS in the past year but had MetS escalation.

#### Individual variables and measurements

**Individual variables** comprised demographic data (sex, age, marital status, education level, occupation, income sufficiency, and family type), perception of community participation, clinical data, and participants’ health behaviors. Data were obtained from the patient clinical information record form (Supplement I) and an interviewing form for older adults with NCDs (Supplement II). Results related to the frequency and percentage of the individual-level factors classified by the hospital levels were shown in Supplement IV.

***The perception of community participation*** was measured by questionnaires developed based on the concept of community participation proposed by Cohen and Uphoff [3]. The interview form assessed older adults’ perceptions of local government involvement in the community’s control of MetS of older adults with NCDs. The interview form contained six questions in a Yes / No format assessing four processes of community participation as follows: participating in decision making (2 items), in the implementation (2 items), in receiving benefits (1 item), in the evaluation of project implementation /activity for controlling MetS in the community (1 item). In the current study, the Content Validity Index (CVI) was 0.86, and Cronbach’s Coefficient alpha was 0.84.

***Comorbidity*** is defined as older adults with NCDs having more than one disease diagnosed according to ICD-10 within the past year of receiving the services at the NCD Plus clinics constituted comorbidity. The instrument used to assess comorbidity was the Charlson Comorbidity Index (CCI) developed by Charlson et al. (2008) [32]. A total comorbidity score ranges from 0 to 42, where a high score means severe comorbidity and a low score means mild comorbidity. This study classified the comorbidity into four groups: no comorbid, mild comorbid, moderate comorbid, and high comorbid. ***Polypharmacy*** is the number of medications patients receive from their treatment, as recorded in the medical records. This study divided polypharmacy into more than or equal to 5 drugs and less than five.

***Health behaviors*** included dietary patterns, physical activities, and medication adherence. ***Dietary patterns*** refer to the quantity, proportion, variety, or mix of foods, beverages, and nutrients in food and the frequency of habitual food consumption. The dietary patterns of older adults with NCDs were assessed with two questions according to the Health Behavior Assessment Tool: 3E2S (eating, exercise, emotion, stop smoking, and stop drinking alcohol) (2018 revised version) by the Division of Health Education, Ministry of Public Health. Cronbach’s Alpha coefficient in the current study was 0.66. The two items were “Controlled the amount of food and the taste of food, not to eat sweet, oily, salty food” and “How often you ate at least half a kilogram of fresh, clean fruits and vegetables daily.” The dietary pattern was scored as practice three days or more/week of both items = 1 point; practice less than three days/week of any item = 0 points. Performing body movements through the work of the skeleton muscles constituted ***Physical activity.*** The item was “Had moderate intensity exercise 30 minutes /time /at least three times per week or at least 150 minutes per week, or had at least 75 minutes for the intensity level”. The physical activity was scored as practice three days or more /week = 1 point; practice less than three days/week = 0 points. ***Medication adherence*** means the cooperation of older adults with the correct NCD medications regularly, continuously taken on time, and in the correct dosage to treat or control underlying disease symptoms according to the doctor’s treatment plan. This study applied the Medication Adherence Scale in Thais (MAST) as an interview form to collect information on drug intake according to the treatment plan of older adults with NCDs over the past month. MAST had a Cronbach’s Alpha Coefficient of 0.71 [33]. The MAST interview form consists of 8 questions; each has six-response answers (0–5 scores); a total score of MAST ranges from 0 to 40. MAST score ≥ 34 means that he/she took medication continuously and consistently or adhered to it.

#### Variables related to health service system and measurement

**Variables related to the health service system** included the hospital level, policy, the NCD Plus clinics’s quality, and the care delivery process. The researchers gathered information from the document and an interview with the heads of the NCD Plus clinics. The questionnaires obtained data from the health service system, as shown in Supplement III.

*Level of hospital* refers to the classification of hospitals according to the service plan policy specifications of the Ministry of Public Health. The hospitals are divided into three levels: First, middle, and high. *Policy* means that the hospital has an action plan for NCD Plus clinics, and MetS control has been planned and is being implemented. *The quality of NCD Plus clinics* means an outcome evaluation of the health service provision of the NCD Plus clinics, which supports NCD care and management to decrease the risk, reduce the disease, and prevent complications from illness for better health improvement. In assessing the quality of NCD Plus clinics, the researcher applied a questionnaire developed by the Bureau of Non-Communicable Diseases, Department of Disease Control, Ministry of Public Health. The questionnaire assessing this aspect contains 21 questions. This study classified the quality of the NCD Plus clinic into five categories: exceptional, excellent, good, fair, and poor. *Process of care delivery* refers to the care process for older adults with NCDs that enables them to control their MetS, including screening (complete screening, partial screening, and no screening whenever the patient comes for a check-up), patterns of health education for self-care (no health education, individual health education with educational materials, and both individual and group health education with educational materials), health behavior monitoring (none, some health behaviors, and all health behaviors related to MetS control). Connecting services to the communities refers to returning patients’ information with MetS to local administration organizations so that they can participate in data analysis and develop corresponding plans. Five questions gathered information on this aspect. This study classified connecting services to the communities into three groups: no, partial, and complete process of connecting services. The questionnaire’s Content Validity Index (CVI) assessing the health system service was 0.90, and Cronbach’s Alpha Coefficient was 0.84.

### Statistical analyses

The frequency and percentages of MetS changes from the initial assessment one year ago were calculated and classified into controlled and uncontrolled MetS. No MetS escalation and MetS decrease to no MetS were classified as a group of controlled MetS. No metS then escalated to have MetS, and still having MetS from the initial assessment was classified as a group of uncontrolled MetS. The control of MetS was calculated at the hospital level as well. The individual and hospital service system factors were considered independent variables influencing MetS control using univariate logistic regression analysis. The odds ratio (OR) and 95% confidence intervals (CI) were estimated for each categorical factor. The individual and hospital service system factors associated with 1-year MetS control with *p* ≤ 0.20 were selected for further analysis. Multilevel logistic regression, a nested model, combines variables at individual and hospital levels. Model 1 analyzed only the dependent variable (1-year MetS control) without any predictor variables and calculated the intraclass correlation coefficient (ICC). Model 2 analyzed only the relationship between 1-year MetS control and the first-level predictor variable (patient level), including sex, age, age, marital status, education, occupation, income sufficiency, type of family, community participation, comorbid, polypharmacy, dietary patterns, physical activities, and medication adherence. Model 3 investigated the relationship between 1-year MetS control and first- and secondary-level predictors (hospital level, policy, NCD Plus clinic quality, screening, health education pattern, health behavior monitoring, connecting service to the community). The log-likelihood ratio (Chi-Square Test) was applied to estimate the first- and secondary-level coefficients for predicting the 1-year MetS control. All data was analyzed using STATA/IC version 16.1.

## Results

The mean age of the study population was 72.9 (SD 5.5) years. Most of the participants were aged 60–69 years (60.8%), were female (58.8%), were married (64.2%), graduated from elementary schools (69.3%), were unemployed, lived in nuclear families (54.0%), and had sufficient income with saving (40.7%). Most participants reflected that the community did not participate in the prevention and control of MetS in the community (44.8%). Approximately 66.0%, 45.3%, and 43.7% of participants took polypharmacy, healthy dietary patterns, and exercise, respectively. Up to 86.5% of participants had medication adherence (Table 1). According to the characteristics of the health service system, it was found that most hospitals had policies to prevent and control MetS. Regarding the quality of NCD Plus clinics, 41.7% of hospitals had a reasonable quality of NCD Plus clinics; 41.7% did MetS screening every time the patient came to receive service. Most Health Education (HE) patterns for MetS control were available in individual, group, and material formats. Most NCD Plus clinics monitored health behaviors for all issues format (83.3%). Most hospitals did complete connecting services to the communities (83.4%) (Table 2).

**Table 1:** Individual characteristics of the participants

Variables	participants	
	**Total number** (***n*** **= 600)**	%
**Sex**		
Male	247	41.2
Female	353	58.8
**Age (year)**		
60–69	365	60.8
70–79	191	31.8
≥ 80	44	7.4
Mean 72.9 (SD. 5.5)		
**Marital status**		
Single	44	7.3
Married	385	64.2
Widowed/Divorced/ Priest	171	28.5
**Education**		
Illiterate	43	7.2
Primary school	416	69.3
Secondary/High school	71	11.8
Diploma/ university	70	11.7
**Occupation**		
Unemployed	324	54.0
Labor-employed	50	8.3
Agriculture	92	15.4
Sales/Business	62	10.3
Retried	72	12.0
**Income sufficiency**		
Sufficiency with saving	244	40.7
Sufficiency with no savings	232	38.7
Insufficiency	69	11.5
No income	55	9.1
**Type of Family**		
Nuclear	292	48.7
Three-generation	243	40.5
Skipped-generation	65	10.8
**Process of Community Participation**		
Not at all	269	44.8
Partly	216	36.0
All processes	115	19.2
**Comorbid burden**		
No-comorbid	9	1.5
mild-comorbid	514	85.7
Moderate-comorbid	70	11.7
high-comorbid	7	1.1
**Polypharmacy**	369	66.0
**Dietary patterns**		
Unhealthy dietary patterns	328	54.7
Healthy dietary patterns	272	45.3
**Physical activity**		
Non-exercise	338	56.3
Exercise	262	43.7
**Medication adherence**	519	86.5

**Table 2:** Characteristics of health service system factors

Variable	Total number (%)	Number (%) Hospital level (*n* = 12)		
	(*n* = 12)	First-level (*n* = 3)	Middle-level (*n* = 5)	High-level (*n* = 4)
**Policy**				
No	1 (8.3)	0 (0.0)	0 (0.0)	1 (25.00)
Yes	11 (91.7)	3 (100.0)	5 (100.0)	3 (75.00)
**Quality of** NCD Plus clinics				
Below basic (poor)	0 (0.0)	0 (0.0)	0 (0.0)	0 (0.0)
Basic (fair)	3 (25.0)	2 (66.7)	1 (20.0)	0 (0.0)
Good	5 (41.7)	0 (0.0)	1 (20.0)	4 (100.0)
Very good	1 (8.3)	0 (0.0)	1 (20.0)	0 (0.0)
Excellent	3 (25.0)	1 (33.3)	2 (40.0)	0 (0.0)
**Screening**				
None	0 (0.0)	0 (0.0))	0 (0.0))	0 (0.0))
Partial screening	7 (58.3)	1 (33.3)	5 (100.0)	1 (25.0)
Completed	5 (41.7)	2 (66.7)	0 ()	3 (75.0)
**Patterns of Health Education (HE)**				
No, HE	1 (8.3)	0 (0.0)	0 (0.0)	1 (25.0)
Individual & material	5 (41.7)	0 (0.0)	3 (60.0)	2 (45.0)
Individual & group & material	6 (50.0)	3 (100.0)	2 (40.0)	1 (25.0)
**Health behavior monitoring**				
None	0 (0.0)	0 (0.0)	0 (0.0)	0 (0.0)
partial issues	2 (16.7)	0 (0.0)	2 (40.0)	0 (0.0)
All issues related to MetS control	10 (83.3)	3 (100.0)	3 (60.0)	4 (100.0)
**Connecting services to the communities**				
None	1 (8.3)	0 (0.0)	0 (0.0)	1 (25.0)
Some services	1 (8.3)	0 (0.0)	0 (0.0)	1 (25.0)
All services (complete)	10 (83.4)	3 (100.0)	5 (100.0)	2 (50.0)

*HE *Health education

*MetS *Metabolic syndrome

*NCD* Non-communicable diseases

Regarding the control of MetS within one year of older adults with NCDs attending NCD Plus clinics, it was found that 24% of participants could control their MetS. Regarding the hospital level, 26% of participants receiving NCD Plus clinic services at high-level hospitals could control their MetS, followed by those at first-level hospitals (24.2%) (Table 3). The results showed that the number of older adults with MetS escalated from 440 at the initial assessment to 456 at the follow-up, marking a 3.6% increase. Specifically, the increase in MetS prevalence among older adults was 7.7% for first-level hospitals, a decrease of 8.0% for middle-level hospitals, and an increase of 4.2% for high-level hospitals. The incidence of new MetS cases in previously unaffected older adults was 16.3% for all levels, 20% for first-level hospitals, 10.3% for middle-level hospitals, and 16.3% for high-level hospitals. Conversely, for older adults initially diagnosed with MetS, only 2.3%, 1%, 3.3%, and 2.3% transitioned to non-MetS status by the second assessment for all levels, first, middle, and high-level hospitals, respectively.

**Table 3:** Frequencies and percentages of MetS control/change within one year of the participants categorized by hospital level

Variable	Total number (%)	Number (%) at Hospital level (*n* = 12)		
	(*n* = 600)	First-level (*n* = 149)	Middle-level (*n* = 151)	High-level (*n* = 300)
**Control of MetS**				
No	456 (76.0)	113 (75.8)	121 (80.1)	222 (74.0)
Yes	144 (24.0)	36 (24.2)	30 (19.9)	78 (26.0)
**MetS changes (pattern)**				
- No MetS to no MetS	134 (22.3)	36 (6.0)	26 (4.3)	72 (12)
- MetS to no MetS	10 (1.7)	1 (0.2)	4 (0.7)	5 (0.8)
- No MetS to MetS	26 (4.3)	9 (1.5)	3 (0.5)	14 (2.3)
- MetS to MetS	430 (71.7)	103 (17.2)	118 (19.7)	209 (34.8)

*MetS *Metabolic syndrome

The univariate logistic regression analysis showed that individual factors, including sex, age group, occupation, income sufficiency, community participation, polypharmacy, dietary patterns, and medical adherence, had a chance of affecting controlled MetS ( *p* < 0.20, as shown in Table 4. These variables were candidates for further multilevel analysis.

**Table 4:** Univariate logistic regression analysis of individual factors influencing 1-year MetS control in older adults with NCDs

Personal factors	OR	SE	z	*p*-value	95%CI	
**Predisposing factors**						
**Sex**						
Male	Reference					
Female	0.37	0.07	-4.68	0.00**	0.24	0.56
**Age group**						
60–69 years	Reference					
70–79 years	1.21	0.28	0.82	0.41	0.76	1.92
≥ 80 years	2.45	0.92	2.39	0.01**	1.17	5.14
**Marital status**						
Single	Reference					
Married	1.02	0.40	0.06	0.94	0.47	2.23
Widowed/Divorced/ Priest	1.03	0.44	0.07	0.94	0.43	2.41
**Education**						
Illiterate	Reference					
Primary school	2.12	1.04	1.54	0.12	0.81	5.58
Secondary/High school	1.60	0.96	0.83	0.40	0.51	5.19
Diploma/university level	1.31	0.92	1.10	0.69	0.33	5.19
**Occupation**						
Unemployed	Reference					
Agriculture	1.69	0.48	1.86	0.06	0.97	2.95
Labor -employed	2.12	0.78	2.05	0.040*	1.03	4.37
Sales	0.74	0.29	-0.73	0.466	0.34	1.63
Retried	0.56	0.19	-1.68	0.107	0.28	1.13
**Income sufficiency**						
Sufficiency with saving	Reference					
Sufficiency with no savings	0.80	0.19	-0.89	0.37	0.49	1.30
Insufficiency	0.92	0.35	-0.20	0.84	0.43	1.96
No income	0.48	0.21	-1.64	0.10	0.20	1.14
**Enabling factors**						
**Type of family**						
Nuclear	Reference					
Three-generation	1.06	0.24	0.28	0.77	0.67	1.68
Skipped-generation households	1.51	0.51	1.21	0.22	0.77	2.96
**Community participation**						
No community participation	Reference					
In some processes	0.72	0.17	-1.33	0.18	0.45	1.16
In all processes	0.63	0.19	-1.51	0.13	0.34	1.14
**Need factor**						
**Comorbid**						
No-comorbid	Reference					
Mild-comorbid	0.83	0.33	-0.46	0.64	0.37	1.84
Moderate-comorbid	0.74	0.30	-0.71	0.47	0.33	1.67
High-comorbid	0.89	0.34	-0.29	0.77	0.41	1.91
**Polypharmacy**						
< 5 drugs	Reference					
≥ 5 drugs	0.46	0.10	-3.52	0.000**	0.30	0.71
**Health behavior**						
**Dietary patterns**						
Unhealthy dietary patterns	Reference					
healthy dietary patterns	1.74	0.36	2.67	0.008**	1.159	2.61
**Physical activity**						
Non-exercise	Reference					
Exercise	1.20	0.25	0.85	0.39	0.78	1.83
**Medication adherence**						
No	Reference					
Yes	2.95	1.11	2.87	0.004**	1.40	6.17

*CI C*onfidence interval, *SE S*tandard error, *OR *Odds ratio

*z* z-test indicates a significant relationship between the hospital service system factor and 1-year MetS control

* Significant at *p*<0.05;** *p*<0.01

The univariate logistic regression analysis showed that health service system factors, including policy related to MetS control, quality of NCD Plus clinics, the pattern of screening, patterns of health education, and health behavior monitoring, had a likelihood to affect MetS control (*p* ≤ 0.20), as shown in Table 5. These variables were candidates for further multilevel analysis.

**Table 5:** Univariate logistic regression analysis of hospital service system factors influencing 1-year MetS control in older adults with NCDs

Hospital Service Factors	OR	SE	z	*p*-value	95%CI	
**Hospital level**						
First-level	Reference					
Middle-level	0.77	0.21	-0.90	0.37	0.44	1.34
High- level	0.92	0.22	-0.31	0.75	0.57	1.49
**Policy**						
No						
Yes	0.45	0.10	-3.44	0.01**	0.18	0.62
**Quality of** NCD Plus clinics						
Excellent	Reference					
Very good	0.38	0.25	-1.44	0.15	0.13	1.36
Good	0.50	0.15	-2.16	0.03*	0.27	0.94
Basic (fair)	0.44	0.15	-2.31	0.02*	0.22	0.88
**Screening**						
Partial screening	Reference					
Completed screening	1.40	0.33	1.39	0.16	0.87	2.25
**Patterns of health education**						
No health education	Reference					
Individual & material	1.61	0.34	2.24	0.02*	1.06	2.46
Individual, group, & material	1(empty)					
**Health behavior monitoring**						
Some issues	Reference					
All issues related to MetS control	1.85	0.48	2.35	0.01**	1.1	3.10
**Connecting services to the communities**						
None	Reference					
Some services	1(empty)					
All services (complete)	0.95	0.34	-0.12	0.90	0.46	1.95

*OR O*dds ratio, *CI *Confidence interval

*SE S*tandard error, *z *z-test indicates a significant relationship between the hospital service system factor and 1-year MetS control

* Significant at *p* < 0.05 ** *p* < 0.01

The results of the multilevel analysis are shown in Table 6. In model 1, the analysis of only the MetS control, the result presented the intraclass correlation coefficient (ICC) with *p* = 0.057; further analysis with multilevel was appropriate for this study because it informed us of the variance of the MetS control attributed to the hospital-level factors [34]. Model 2, the analysis of only individual factors, found that females, age ≥ 80 years, labor employment occupation, polypharmacy ≥ 5 types of drugs, healthy dietary patterns, and medication adherence compared with the reference groups affected MetS control (*p* < 0.05; intercept = 0.25). Model 3 contained both individual and hospital-level factors in the analysis and found that the same individual-level factors and hospital-level factors, only the provision of MetS screening services for patients every time they came to receive the service could significantly affect MetS control among older adults with NCDs receiving services at NCD Plus clinics of the hospitals (*p* < 0.05; intercept = 0.20). The result of the model 3 analysis presented the performance of the fixed effect model with a Log-likelihood (LL) value higher than that of other models. The different service system factors at the Hospital level affected MetS control among older adults with NCDs.

On the individual level, the results revealed that the risk of control MetS increased with individual factors, including sex and polypharmacy. Female older adults had a 66% decrease in odds of controlling their MetS compared to males (OR = 0.34 95%CI 0.22-0.53); *P* < 0.001), and those who taking ≥ 5 types of drugs had a 54% decrease in odds of controlling their MetS (OR = 0.46, 95%CI 0.30-0.71; *p* < 0.001) compared to those who taking lower types of drugs. Concerning the age group, the older adults age ≥ 80 years have a chance to control MetS increased by 2.38 folds (OR = 2.38 95%CI 1.12–5.05; *p* < 0.05) compared to the age 60–69 years. In the occupation group, the results were contrary; the labor-employed increased their MetS control by 2.14 folds compared to non-employed older adults (OR = 2.14 95%CI 1.03–4.42; *p* < 0.05). Meanwhile, the retried older adults increase the chance of uncontrolled 1-year MetS compared to the unemployed (OR = 0.48 95%CI 0.23–0.99; *p* < 0.05). In the hospital level, the fixed effect of hospital service level showed that complete screening for MetS conditions enhances the controlled 1-year MetS by 1.76 folds compared to partial screening (OR = 1.76 95%CI 1.06–2.97; *p* < 0.05). Meanwhile, no other health service system factors were shown to be significant factors affecting MetS control.

**Table 6:** Estimated odds ratio and variance components for the multilevel logistic regression models

Variables	Model 1	Model 2		Model 3	
		Odds ratio (95%CI)	*p*-value	Odds ratio (95%CI)	*p*-value
**Fixed effect**					
**Patient-level**					
Intercept	-1.12 (-1.45, − 0.79)	0.25 (0.10, 0.61)	0.002^**^	0.20 (0.08, 0.48)	0.000^***^
**Sex**					
Male (Reference)					
Female		0.35 (0.23, 0.54)	0.000^***^	0.34 (0.22, 0.53)	0.000^***^
**Age**					
60–69 years (Reference)					
70–79 years		1.13 (0.70, 1.81)	0.604	1.13 (0.70, 1.82)	0.598
≥80 years		2.38 (1.12, 5.07)	0.024^*^	2.38 (1.12, 5.05)	0.023^*^
**Occupation**					
Unemployed (Reference)					
Agriculture		1.61 (0.91, 2.85)	0.101	1.59 (0.90, 2.82)	0.106
Labor-employment		2.14 (1.03, 4.47)	0.041^*^	2.14 (1.03, 4.42)	0.039^*^
Sales		0.73 (0.33, 1.62)	0.446	0.68 (0.30, 1.51)	0.346
Retried		0.54 (0.26, 1.10)	0.092	0.48 (0.23, 0.99)	0.050^*^
**Polypharmacy**					
< 5 types of drugs (Reference)					
≥ 5 types of drugs		0.48 (0.30, 0.75)	0.001^***^	0.46 (0.29, 0.71)	0.001^***^
**Dietary patterns**					
Unhealthy dietary patterns (Reference)					
Healthy dietary patterns		1.61 (1.05,2.47)	0.026^*^	1.61 (1.06,2.46)	0.025^*^
**Medication adherence**					
No (Reference)					
Yes		3.10 (1.47, 6.53)	0.003^**^	3.18 (1.51, 6.70)	0.002^**^
**Hospital-level**					
**Screening for MetS**					
Partial screening (Reference)					
Complete screening				1.76 (1.06, 2.92)	0.028^*^
**Random effect** **ICC**	5.76%	5.22%		2.40%	
**Log-likelihood (LL)**	-326.589	-294.441		-292.451	
** σ** ^**2**^_**µ**_	-1.12	0.207		0.164	
** σ** ^**2**^ **e**	0.201	0.137		0.050	

*CI C*onfidence interval, *ICC *Intraclass correlation coefficient

*Significant at *p* < 0.05 **Significant at *p* < 0.01 ***Significant at *p* < 0.001

## Discussion

In this study, 24% of older adults with NCDs could control MetS within one year, and 76% could not. The count of MetS escalation, from the initial assessment to 1-year follow-up, varied according to the level of the hospitals. The transition from MetS to non-MetS status was rare in older adults with NCDs. This finding is consistent with a study in Iran, which found that 51.7% of older adults could not control their MetS [35]. On the contrary, a previous study among older adults with diabetes mellitus, hypertension, or both received primary care in a health district of Brazil based on the chronic care model. After follow-up of a family health team for at least one year, MetS control and blood pressure levels were at 74.3% and 54.3% [36]. According to Healthy Aging, the community program training for Mexican older adults on the control of MetS during six months (2 h per week) reported that of those who participated in the program, 28% maintained the diagnosis of MetS, compared to 83% of those who participated in the control group maintained the diagnosis of MetS [3]. The proportion of older adults who can control MetS after time has passed differed depending on the health service received, program implementation, and personal factors [3].

Among health service system factors, complete screening for MetS influenced 1-year MetS control among older adults with NCDs receiving service in the NCD Plus clinic. The health service system presents the inequality of structure and service delivery process among the hospitals relative to the control of MetS. Compared with the previous multilevel study based on socioeconomic factors among the population in western China, the number of beds in medical institutions per 1000 people and urbanization rate positively affected MetS. In contrast, with the number of doctors in healthcare institutions per 1000 people, the risk of MetS was relatively low. Both studies differed in the health outcome regarding 1-year control of MetS and MetS diagnosis. However, the findings suggest the importance of health resources and care processes in the health service system.

The health service delivery by providing a complete screen of MetS whenever patients attend NCD Plus Clinic affects 1-year control of MetS. Screening is the search for risks, problems, and causes of health problems. The data results are used to plan and provide further treatment [17]. A complete MetS screening consisted of weight, height, waist circumference, blood pressure measurements, and blood tests for triglyceride, HDL cholesterol, and blood sugar levels. The screening process included explaining the examination and diagnosis results of MetS to older adults every time they came to receive the service. The complete screening increased the chance of controlling MetS of older adults with NCDs. This finding is consistent with a study in Egypt where healthcare providers screen MetS to older adults who come to receive services at the outpatient departments of the university hospitals every time. Regular attendance reduced the number of older adults with MetS statistically and significantly [37]. Consistent with the NCD Plus Clinic results in hospitals in Thailand in 2017–2019, which service delivery to risk groups and patients with diabetes and hypertension through screening, data feedback, and continuous self-care monitoring, helping to reduce the incidence of diabetes and hypertension in risk groups and reduce cardiovascular disease (CVD), and chronic kidney disease (CKD) in patients [38, 39]. The complete screening helped older adults receive comprehensive care that better met their problems and needs [15].

The individual factors affect the one-year control of MetS within the hospital service level, including sex, age, occupation, polypharmacy, dietary patterns, and medication adherence. Regarding predisposing factors, females are a risk factor, whereas ≥ 80 years is a protective factor for the one-year control of MetS. This finding is consistent with a previous study in Thailand, where the older female adults had a 9.83-fold lower likelihood of MetS control [40]. In addition, a study in China reported that older female adults had a 2.49-fold odds of having MetS greater [10]. Two possible reasons are: first, estrogen levels reduce, and androgen levels elevate in elderly females during menopause, decreasing basal metabolic rate. Along with body fat distribution abnormality, their opportunity to control MetS decreases [41].

The older adults aged ≥ 80 have a chance to control MetS increased compared to the age 60–69 years. This result is consistent with a study’s result in China. The Chinese study found that older adults aged 70 and older controlled their MetS better than younger adults [10]. Since both free fat and muscle mass in the 70-year-old and older elderly decrease equally, there is a decrease in fat accumulation, resulting in MetS control [42]. In addition, most of the older adults do not have teeth or wear dentures, making chewing inconvenient and requiring soft and easily digestible food. Moreover, the function of the gastrointestinal tract slows down and causes long digestion, so they eat less, resulting in controlled MetS [43].

Regarding occupation, a predisposing factor is that labor-employed and retried older adults control their MetS more than non-employed older adults. Correspondingly, a study in China found that older adults working in agriculture controlled MetS better than those not engaged in agriculture. This finding is consistent with a study in Malaysia that found that retired older adults with a good understanding of healthy lifestyle knowledge can directly influence control MetS [44]. The occupation involves physical activity, thereby increasing metabolism and controlling MetS [10].

The older adults taking polypharmacy had a 54% decrease in odds of controlling their MetS. Polypharmacy may cause older adults with NCDs to be fed up with many medications and not want to take medications continuously. As a result, they cannot control uncontrolled blood sugar, lipid, BP, and MetS. A supporting study in Brazil found that age, polypharmacy, and use of inappropriate medication may lead to drug interactions among older adults with Mets [23]. Therefore, health teams, including doctors and nurses, should monitor the patient’s medication intake, not only its completeness and continuity but also the types of drugs the patients currently take to assess polypharmacy and use this information in planning further treatments.

Medication adherence by older adults is 3.18 times more likely to control MetS than insufficient medication adherence. Therefore, medication adherence for blood sugar, blood pressure, and lipid levels influenced MetS control in older adults with NCDs [45–47].

Dietary patterns influenced MetS control. Older adults with NCDs who followed healthy diet patterns were 1.61 times more likely to control MetS than those who followed unhealthy dietary patterns. Eating healthy dietary patterns prevents MetS in older adults with NCDs. A nutritious diet is more suitable for energy use than fat and blood sugar accumulation [24].

Therefore, to control the MetS of older adults with NCDs, the health service needs to emphasize individual factors by paying specific attention to females, the labor-employed, and the retired, promoting medication adherence, healthy dietary patterns, and advising on polypharmacy. In addition, healthcare providers must accentuate the provision of health services, with complete and continuous MetS screening for older adults with NCDs, to control MetS and the complications that may occur.

### Strengths and limitations

This study provides evidence of MetS control among older adults with NCDs who attend NCD Plus clinics of the hospitals in a year. The study is the first in Thailand to employ multi-factor prediction analysis, in which patient- and hospital-level factors play an essential role in controlling the MetS of older adults with NCDs. The study provides crucial information for planning health service provision in the NCD Plus clinics to control MetS in older adults with NCDs. However, this study has some limitations. The participants in this study had at least one of three NCDs: hypertension, diabetes, and hyperlipidemia. The characteristics of the population are quite different. In future research, it would be better to study specific diseases or conditions to obtain appropriate guidelines for controlling MetS specific to the disease. This study is hospital-based. Therefore, the study results differed from those in the general elderly population. These results only occurred in older adults aged more than 60 years who were diagnosed with hypertension, diabetes, or hyperlipidemia for at least one year. One of this study’s limitations is that the medical records of the past year may not be complete. The patients may take medications from other places. As a result, future studies should expand to the group of older adults with NCDs and other diseases for a more comprehensive and precise overview of the service provision to cover all older adult groups.

## Conclusions

NCDs Plus clinics that provide complete screening for MetS make it possible to enhance their effectiveness in reducing the proportion of older adults with MetS. This screening involves aiding those already with MetS in reverting to non-MetS status and preventing those without MetS from developing it. Furthermore, older adults who are aged ≥ 80 years, have labor-employment, healthy dietary patterns, and medication adherence were beneficial to control MetS within one year. In contrast, those who are female, are retired, and take multiple medications have an elevated risk of not achieving one-year MetS control. The insights gained from such an analysis could be instrumental in pinpointing the resources necessary to bolster the efficacy of NCD Plus clinics.

## Electronic supplementary material

Below is the link to the electronic supplementary material.

Supplementary Material 1.

Supplementary Material 2.

Supplementary Material 3.

Supplementary Material 4.

## Data Availability

The datasets generated and analyzed during the current study are not publicly available due to the data protection declaration used but are available from the corresponding author upon reasonable request.

## Abbreviations

MetS: Metabolic syndrome
NCDs: Non-communicable diseases
CCM: Chronic Care Model
BM: Behavioral model of health service uses
PMQA: Public Sector Management Quality Award
IDF: International Diabetes Foundation
HDC: Health Data Center
CCI: Charlson Comorbidity Index
CVI: Content Validity Index
OR: Odds ratio
LR: Logistic regression
MAST: Medication Adherence Scale in Thais
ICD: International Statistical Classification of Disease and Related Health Problem
