# Supplementary material for: Examining determinants of control of metabolic syndrome among older adults with NCDs receiving service at NCD Plus clinics: multilevel analysis
Source: BMC Health Serv Res. 2024 Sep 27;24:1118. doi: 10.1186/s12913-024-11562-3 (PMC11429379; doi:10.1186/s12913-024-11562-3)
Supplement: Supplementary file 1 — Supplementary Material 1. [file 12913_2024_11562_MOESM1_ESM.docx]

Record code....................................

**Patient Clinical Information Record Form from OPD Card**

**Instruction**: Please fill in the blanks as truthfully as possible.

1. Weight……………… kgs.
2. High………………... cms
3. BMI…………………………. kg/m ^2^
4. The number of medications the patient currently takes ….

( ) 1 medicine ( ) 4 medicines

( ) 2 medicines ( ) 5 medicines

( ) 3 medicines ( ) More than 5 medicine, specify the number ……..medicines.

1. Clinical symptoms and the diagnosis results of metabolic syndrome

| **Latest test results**  **(dd/mm/yyyy) ..../.../....** | **Results** | **Previous results within 1 year**  **(dd/mm/yyyy) ..../.../....** | **Result** |
| --- | --- | --- | --- |
| **-** Waist circumference |  | **-** Waist circumference |  |
| **-** Triglycerides or taking hypolipidaemic agents |  | **-** Triglycerides or taking hypolipidaemic agents |  |
| -HDL cholesterol or taking hypolipidaemic agents |  | -HDL cholesterol or taking hypolipidaemic agents |  |
| -Blood pressure or taking antihypertensive drugs |  | -Blood pressure or taking antihypertensive drugs |  |
| **-** Fasting glucose or taking diabetes medications |  | **-** fasting glucose or taking taking diabetes medications |  |
| Diagnosis results |  | Diagnosis results |  |

1. Results of metabolic syndrome control within 1 year ( ) Controlled ( ) Uncontrolled

**The Charlson Comorbidity Index (CCI ) Assessment Form**

**Objectives:** The Charlson Comorbidity Index ( CCI ) assessment form is a tool used to assess the comorbidity burden of patients to find relationships between comorbidities and metabolic syndrome in older adults with chronic non-communicable diseases (NCDs).

**Instruction**: Please put **🗸** into ❒ that corresponds to the comorbidity burden of the older adult with NCDs who has received services in the NCD Clinic Plus. The information on comorbidities can be obtained from the database in the patient's medical record. This information is assessed for patient receiving the services in the previous 1 year.

| **Conditions** | | | **Assigned weights for disease** |
| --- | --- | --- | --- |
| CCI-1 | cerebrovascular disease | ❒ | 1 |
| CCI-2 | congestive heart failure | ❒ | 1 |
| CCI-3 | COPD/asthma | ❒ | 1 |
| CCI-4 | dementia | ❒ | 1 |
| CCI-5 | depression | ❒ | 1 |
| CCI-6 | diabetes without end organ | ❒ | 1 |
| CCI-7 | Hypertension | ❒ | 1 |
| CCI-8 | mild liver disease | ❒ | 1 |
| CCI-9 | myocardial infarct | ❒ | 1 |
| CCI-10 | peripheral vascular disease | ❒ | 1 |
| CCI-11 | rheumatic disease | ❒ | 1 |
| CCI-12 | ulcer disease | ❒ | 1 |
| CCI-13 | use of warfarin | ❒ | 1 |
| CCI-14 | hemiplegia | ❒ | 2 |
| CCI-15 | moderate or severe renal disease | ❒ | 2 |
| CCI-16 | diabetes with end organ damage | ❒ | 2 |
| CCI-17 | any tumor | ❒ | 2 |
| CCI-18 | leukemia | ❒ | 2 |
| CCI-19 | lymphoma | ❒ | 2 |
| CCI-20 | skin ulcer/cellulitis | ❒ | 2 |
| CCI-21 | moderate or severe liver disease | ❒ | 3 |
| CCI-22 | metastatic solid tumor | ❒ | 6 |
| CCI-23 | AIDS | ❒ | 6 |
| Total score | | |  |
