# Supplementary material for: Examining determinants of control of metabolic syndrome among older adults with NCDs receiving service at NCD Plus clinics: multilevel analysis
Source: BMC Health Serv Res. 2024 Sep 27;24:1118. doi: 10.1186/s12913-024-11562-3 (PMC11429379; doi:10.1186/s12913-024-11562-3)
Supplement: Supplementary file 2 — Supplementary Material 2. [file 12913_2024_11562_MOESM2_ESM.docx]

Interview form code..................................

**Interview Form for Older Adults with NCDs Receiving Services at NCD Clinic Plus**

This interview form contains four parts: part 1: General information, part 2: Self-care behavior, part 3: medication adherence, and part 4: Perception towards Community participation in Controlling metabolic syndrome for older adults with NCDs in the community.

**Part 1 General information**

**Instruction:** In each question, the interviewer asks the older adult and puts 🗸in (...) that corresponds to the answer given by the older adult. Please ask the older adult all questions.

1. Sex ( ) Male ( ) Female

2. Age ......... years

3. Marital status

( ) Single ( ) Married ( ) Divorced ( ) Separated ( ) Widowed

4. Education level

( ) Not educated ( ) Associate's degree/Vocational certificate

( ) Primary education ( ) Bachelor's degree

( ) Junior high school ( ) Master's degree

( ) High School/Vocational Certificate ( ) Doctoral degree

5. Main occupation

( ) Farming, rice farming, gardening ( ) General/labor employment

( ) Selling /business ( ) Not working/Staying at home as a housewife/Househusband

( ) Pensioner ( ) Others........................

6. Sufficiency of income

( ) Sufficient, with some saving ( ) Sufficient, without saving

( ) Insufficient ( ) Insufficient, with debt

7. What is the type of family living today?

( ) Living alone ( ) Husband-wife family (no children)

( ) Family of parents and children

( ) Three-generation family (grandparents, children, grandchildren)

( ) Single-parent family

( ) Skipped-generation family (grandparents, grandchildren)

( ) Family having persons who are not relatives

( ) Others, specify …………………………………………….

**Part 2 Health behaviors**

**Instruction:** In each question, the interviewer asks the older adult about the health behavior practice over the past 1 year and puts 🗸in the box that corresponds to the answer given by the older adult. Please ask the older adult all questions.

| **Practices** | **Average frequency of practice /week** | | | | |
| --- | --- | --- | --- | --- | --- |
|  | 6-7 days | 4-5 days | 3 days | 1-2 days | Not practice |
| 1. Controlled the amount of food and the taste of food, not to eat sweet, oily, salty food |  |  |  |  |  |
| 2. How often you ate at least half a kilo of fresh, clean fruits and vegetables daily |  |  |  |  |  |
| 3. Had moderate intensity exercise 30 minutes /time /at least 3 times per week or at least 150 minutes per week, or had at least 75 minutes for the intensity level? |  |  |  |  |  |

**Part 3: Medication adherence**

**Instruction: In** each question, the interviewer asks the older adult **about medication adherence** and puts × in the box that corresponds to the answer given by the older adult. Please ask the older adult all questions.

| **Item** | **Question** | **Times/month** | | | | | |
| --- | --- | --- | --- | --- | --- | --- | --- |
| **Over the past 1 month,…** | |  |  |  |  |  |  |
| 1 | How often have you forgotten to take medicine? (You have missed taking medicine for some meals) | More than 15 | 10-15 | 6-9 | 3-5 | 1-2 | Never |
| 2 | How often have you changed the medicine dosage based on your needs (taking more or less than you should)? | More than 15 | 10-15 | 6-9 | 3-5 | 1-2 | Never |
| 3 | How often have you stopped taking your medicine? | More than 15 | 10-15 | 6-9 | 3-5 | 1-2 | Never |
| 4 | How often have you not taken your medicine on time ( (taking medicine more than 1 hour before or after your regular medicine-taking time)? | More than 15 | 10-15 | 6-9 | 3-5 | 1-2 | Never |
| 5 | How often have you not taken all of your medicines? | More than 15 | 10-15 | 6-9 | 3-5 | 1-2 | Never |
| 6 | How often have you missed taking your medicine at every meal, such as forgetting to take medicine or to bring medicine to take during your work or while traveling long distances? | More than 15 | 10-15 | 6-9 | 3-5 | 1-2 | Never |
| 7 | How often have you not been to your doctor's appointment (you have missed an appointment or postponed a doctor's appointment)? | very often | often | sometimes | few | seldom | never |
| 8 | How often have you missed your medicine and not taken it because of not coming to see the doctor by appointment? | very often | often | sometimes | few | seldom | never |

**Part 4: Perception towards community participation in controlling metabolic syndrome for older adults with NCDs in the community**

**Instruction**: In each question, the interviewer asks the older adult about **his**/**her perception towards community participation in controlling metabolic syndrome for older adults with NCDs in the community over the past 1 year** and puts 🗸in the box that corresponds to the answer given by the older adult. Please ask all questions. In collecting information on this part, please use the term ‘abdominal obesity’ instead of ‘ metabolic syndrome.’

| **Item** | **Community participation in controlling abdominal obesity of older adults with NCDs in the community** | **Participation** | |
| --- | --- | --- | --- |
|  |  | **Yes** | **No** |
| **Participation in decision-making** | | | |
| Over the past 1 year… | | | |
| 1 | The local administrative organization at the subdistrict/ municipality has participated in village meetings concerning the problem identification and causes of abdominal obesity in older adults with NCDs. |  |  |
| 2 | The local administrative organization at the subdistrict/ municipality has participated in village meetings concerning project planning or activities to control abdominal obesity in older adults with NCDs. |  |  |
| **Participation in implementation** | | | |
| 3 | The local administrative organization at the subdistrict/ municipality participated in providing information about controlling abdominal obesity in older adults with NCDs in the village. |  |  |
| 4 | The local administrative organization at the subdistrict/ municipality has supported a place or equipment for controlling abdominal obesity in the village, such as sport fields, exercise machines, weighting machines, blood pressure machines, etc. |  |  |
| **Participation in receiving benefits** | | | |
| 5 | The local administrative organization at the subdistrict/ municipality has participated in receiving benefits from activities or projects to control abdominal obesity in the community. For example, older adults are more satisfied with the work of the Subdistrict/ Municipality Administrative Organization etc. |  |  |
| **Participation in project evaluation** | | | |
| 6 | The local administrative organization at the subdistrict/ municipality has followed up, evaluated, and presented the implementation/activity results of controlling abdominal obesity in older adults with NCDs to inform people in the community through village meetings or broadcasting towers. |  |  |
