# Supplementary material for: Examining determinants of control of metabolic syndrome among older adults with NCDs receiving service at NCD Plus clinics: multilevel analysis
Source: BMC Health Serv Res. 2024 Sep 27;24:1118. doi: 10.1186/s12913-024-11562-3 (PMC11429379; doi:10.1186/s12913-024-11562-3)
Supplement: Supplementary file 3 — Supplementary Material 3. [file 12913_2024_11562_MOESM3_ESM.docx]

Questionnaire code....................................

**Questionnaire about the health service system for controlling metabolic syndrome**

**at NCD Clinic Plus**

**Instruction:** Please put 🗸in ( ) or fill in the blanks as close to the truth as possible.

1. Type of hospital ( ) Community hospital ( F1 ) ( ) Community hospital ( F2 )

( ) General hospital ( M1 ) ( ) General hospital ( M2 )

( ) Regional Hospital ( A )

1. Size of hospital, number of beds……….
2. Type of clinics providing services

( ) NCD Clinic ( ) Hypertension Clinic

( ) Diabetes Clinic ( ) Quality elderly clinic

( ) Hypertension and Diabetes Clinic ( ) other ……………………………

4. Does your hospital have the following policies regarding the metabolic syndrome (MetS) control for older adults?

| **Item** | **Policy** | **Yes** | **No** |
| --- | --- | --- | --- |
| 1 | Establishing policies and goals to reduce risk factors of MetS. |  |  |
| 2 | Promote understanding about MetS control policy among hospital workers. |  |  |
| 3 | Having an action plan/activities to control MetS . |  |  |
| 4 | Setting the goals of the action plan/activities and success indicators of MetS control. |  |  |
| 5 | Specifying a date, scheduling, and identifying working team for MetS control service |  |  |
| 6 | Providing enough tools, equipment, and suitable place for providing MetS control services |  |  |
| 7 | Evaluating the implementation of the action plan/activities and success indicators in MetS control. |  |  |
| 8 | Reviewing the evaluation results and improving the implementation of MetS control |  |  |

5. The NCD Clinic provides screening services for MetS to older adults with NCDs. The service activities include weighing, measuring height, measuring waist circumference, checking blood pressure levels/taking antihypertensive drugs, checking blood sugar levels/taking diabetes drugs, and checking blood lipid profiles/taking antihyperlipidemic drugs, explaining the test results and MetS diagnosis to older adults with NCDs. Please select one of the following service patterns that actually describes how your clinic has provided to older adults with NCDs.

( ) Complete screening: the clinic provides every service activity every time an older adult with NCDs comes to receive the service.

( ) Partly screening: the clinic provides some service activities every time an older adult with NCDs comes to receive the service.

( ) One time complete screening per year

( ) No screening was done at all.

6. Does your clinic provide knowledge (health education) of controlling metabolic syndrome to older adults with NCDs? If yes, which method for selecting or dividing the older adults into groups to obtain the knowledge from personnel at this clinic?

( ) No, there is no provision of knowledge services (skip to question 9 )

( ) Yes, health educational service is provided by arranging groups of older adults as follows.

( ) Individual

( ) Subgroups as follows:

( ) Group with metabolic syndrome

( ) High risk group (Waist circumference exceeds the criteria and 1 abnormal clinical symptom)

( ) Low risk group (normal waist circumference and 1-4 abnormal clinical symptoms)

( ) Large group (including normal, at-risk older adults, and those with MetS)

7. At each time of health education, how your clinic specifies health education content about controlling metabolic syndrome for older adults with NCDs?

( ) Integrating knowledge of compliance with the principle of 3E2S (eating, exercise, emotion, stop smoking, and stop drinking).

( ) Depending on the topic/content the clinic has determined daily/weekly/monthly

( ) Emphasizing on providing knowledge according to the problems of older adults who come to receive services.

( ) Other..................................

8. How your clinic provide knowledge of controlling MetS to older adults with NCDs each time they come to the service?

( ) Brochure ( ) Watching video

( ) LINE application ( ) other..................................

9. Does your clinic monitors following health behaviors to control MetS in older adults with NCDs?

| **Health behavior monitoring** | **Yes** | **No** |
| --- | --- | --- |
| 1. Eating habit |  |  |
| 2. Exercise method |  |  |
| 3. Stress management |  |  |
| 4. Reducing, quitting, and avoiding smoking |  |  |
| 5. Reducing, quitting, and avoiding drinking alcohol |  |  |
| 6. Taking medicine according to treatment plan |  |  |
| 7. Problems and obstacles in following the treatment plan to prevent and control MetS |  |  |

10. Does your clinic provide following community-connected health services regarding MetS control in older adults with NCDs?

| **Item** | **Providing community-connected services** | **Yes** | **No** |
| --- | --- | --- | --- |
| 1 | Returning analyzed data on patients with MetS to local administrative organizations to be used to control and reduce MetS in the community. |  |  |
| 2 | Together with local government organizations, you clinic has analyzed data and developed plans/ projects/activities to prevent factors affecting the MetS. |  |  |
| 3 | Supporting local government organizations to develop and use public policies to control MetS in the community |  |  |
| 4 | Together with local government organizations, your clinic has implemented projects/ activities to support MetS control in the community, for example, adjusting the environment conducive to disease prevention and control and being a mentor for village health volunteers to perform MetS screening in people in the community. |  |  |
| 5 | Together with local government organizations, your clinic has followed up and evaluated projects/ activities in controlling MetS in the community continuously. |  |  |

11. Your clinic has the results of evaluating the quality of NCD Clinic plus services in 2020 from outside agencies. (Disease Control Office / Provincial Public Health Office)

How is it?

| **list** | **Quality assessment results (score)** |
| --- | --- |
| **Quality development process** | |
| Component 1 : Direction and policy |  |
| Component 2 : Information system |  |
| Component 3 Adjusting the system and service process |  |
| Component 4 : Self-management support system |  |
| Component 5 : Decision support system |  |
| Component 6 : Providing community link services |  |
| **Total quality development process scores ( 50 points)** |  |
| **Regarding the results of service indicators, 12 indicators** | |
| Indicator 1 : Percentage of monitoring of groups suspected of having diabetes. |  |
| Indicator 2 : Percentage of diabetic patients who have had their LDL cholesterol tested and have LDL values. |  |
| Indicator 3 : Percentage of diabetic patients whose blood sugar levels are well controlled. |  |
| Indicator 4 : Percentage of diabetic patients with blood pressure less than 140/90 mmHg. |  |
| Indicator 5 : Percentage of diabetic patients who are obese [ BMI ≥25 kg/sq m] Decreased from the previous fiscal year. |  |
| Indicator 6 : Percentage of acute complications. in diabetic patients |  |
| Indicator 7 : The percentage of new diabetes patients decreased. |  |
| Indicator 8 : Percentage of monitoring of groups suspected of having high blood pressure. |  |
| Indicator 9: Percentage of hypertensive patients whose blood pressure levels are well controlled . |  |
| Indicator 10 : Percentage of patients with diabetes and/or high blood pressure. who have been searched for and screened for chronic kidney disease |  |
| Indicator 11 : Percentage of patients with diabetes and/or high blood pressure who have CVD Risk ≥20% during the 1st and 2nd quarters and have CVD Risk reduced to |  |
| Indicator 12 : Percentage of diabetic patients and/hypertension with CKD 3-4 can delay the decline of eGFR as targeted. |  |
| **Total scores for service indicator results** **(50 points)** |  |
| **Results of evaluating the quality of NCD Clinic plus services (100 points)** |  |
| **Assessment result level** |  |
