# Supplementary material for: Examining determinants of control of metabolic syndrome among older adults with NCDs receiving service at NCD Plus clinics: multilevel analysis
Source: BMC Health Serv Res. 2024 Sep 27;24:1118. doi: 10.1186/s12913-024-11562-3 (PMC11429379; doi:10.1186/s12913-024-11562-3)
Supplement: Supplementary file 4 — Supplementary Material 4. [file 12913_2024_11562_MOESM4_ESM.docx]

**Supplement IV**

**Additional information for understanding the reported results.**

**Table 1** Demographic Characteristics of the Participants Categorized by Hospital Level (n=600)

| **Characteristics** | | **Hospital level** | | | | | | | | | | **Total**  **( n=600 )** | | | |
| --- | --- | --- | --- | --- | --- | --- | --- | --- | --- | --- | --- | --- | --- | --- | --- |
|  |  | **First-level** | | | **Middle-level** | | | **High- level** | | | |  |  |  |  |
|  |  | **Count** | **%** | **Count** | | | **%** | | **Count** | | **%** | | **Count** | | **%** |
| **Participants** | | 149 | 24.8 | | 151 | 25.2 | | 300 | | 50.0 | | 600 | | 100.0 | |
| **Sex** | |  |  | |  |  | |  | |  | |  | |  | |
|  | Male | 50 | 33.6 | | 73 | 48.3 | | 124 | | 41.3 | | 247 | | 41.2 | |
|  | Female | 99 | 66.4 | | 78 | 51.6 | | 176 | | 58.7 | | 353 | | 58.8 | |
| **Age group** | |  |  | |  |  | |  | |  | |  | |  | |
|  | 60-69 | 87 | 58.4 | | 101 | 66.9 | | 177 | | 59.0 | | 365 | | 60.8 | |
|  | 70-79 | 47 | 31.5 | | 44 | 29.1 | | 100 | | 33.3 | | 191 | | 31.8 | |
|  | ≥80 | 15 | 10.1 | | 6 | 4.0 | | 23 | | 7.7 | | 44 | | 7.4 | |
| **Marital status** | |  |  | |  |  | |  | |  | |  | |  | |
|  | Single | 10 | 6.7 | | 9 | 6.0 | | 25 | | 8.3 | | 44 | | 7.3 | |
|  | Married | 98 | 65.8 | | 94 | 62.3 | | 193 | | 64.3 | | 385 | | 64.2 | |
|  | Widowed/Divorced/ Priest | 41 | 27.5 | | 48 | 31.8 | | 82 | | 27.3 | | 171 | | 28.5 | |
| **Education** | |  |  | |  |  | |  | |  | |  | |  | |
|  | Illiterate | 10 | 6.7 | | 15 | 9.9 | | 18 | | 6.0 | | 43 | | 7.2 | |
|  | Primary school | 111 | 74.5 | | 108 | 71.5 | | 197 | | 65.7 | | 416 | | 69.3 | |
|  | Secondary/High school | 20 | 13.4 | | 22 | 14.6 | | 29 | | 9.7 | | 71 | | 11.8 | |
|  | Diploma/ university | 8 | 5.4 | | 6 | 4.0 | | 56 | | 18.7 | | 70 | | 11.7 | |
| **Occupation** | | | | |  |  | |  | |  | |  | |  | |
|  | Unemployed | 91 | 61.1 | | 93 | 61.6 | | 140 | | 46.7 | | 324 | | 54.0 | |
|  | Employed | 9 | 6.0 | | 18 | 11.9 | | 23 | | 7.7 | | 50 | | 8.3 | |
|  | Agriculture | 23 | 15.4 | | 19 | 12.6 | | 50 | | 16.7 | | 92 | | 15.4 | |
|  | Sales/Business | 15 | 10.1 | | 11 | 7.3 | | 36 | | 12.0 | | 62 | | 10.3 | |
|  | Retried | 11 | 7.4 | | 10 | 6.6 | | 51 | | 17 | | 72 | | 12.0 | |
| **Income sufficiency** | | | | | | | | | | | | | | | |
|  | Sufficiency with saving | 60 | 40.3 | | 61 | 40.4 | | 123 | | 41.0 | | 244 | | 40.7 | |
|  | Sufficiency with no savings | 48 | 32.2 | | 59 | 39.1 | | 125 | | 41.7 | | 232 | | 38.7 | |
|  | Insufficiency | 16 | 10.7 | | 17 | 11.3 | | 36 | | 12.0 | | 69 | | 11.5 | |
|  | No income | 25 | 16.8 | | 14 | 9.3 | | 16 | | 5.3 | | 55 | | 9.1 | |

**Table 2** Types of Family of the Participants Categorized by Hospital Level (n=600)

| **Type of Family** | | **Hospital Level** | | | | | | **Total**  **( n=600 )** | |
| --- | --- | --- | --- | --- | --- | --- | --- | --- | --- |
|  |  | **First-level**  **(n=149)** | | **Middle-level**  **(n=151)** | | **High- level**  **(n=300)** | |  |  |
|  |  | **Count** | **%** | **Count** | **%** | **Count** | **%** | **Count** | **%** |
|  | Nuclear | 75 | 50.3 | 83 | 55.0 | 134 | 44.7 | 292 | 48.7 |
|  | Three-generation | 51 | 34.2 | 52 | 34.4 | 140 | 46.7 | 243 | 40.5 |
|  | Skipped-generation | 23 | 15.4 | 16 | 10.6 | 26 | 8.7 | 65 | 10.8 |

**Table 3** Perception of the Participants on Community Participation in the Control of Metabolic Syndrome Categorized by Hospital Level (n=600)

| **Process of Community Participation** | | **Hospital Level** | | | | | | **Total**  **( n=600 )** | |
| --- | --- | --- | --- | --- | --- | --- | --- | --- | --- |
|  |  | **First-level**  **(n=149)** | | **Middle-level**  **(n=151)** | | **High- level**  **(n=300)** | |  |  |
|  |  | **Count** | **%** | **Count** | **%** | **Count** | **%** | **Count** | **%** |
|  | **Decision making** |  |  |  |  |  |  |  |  |
|  | No | 109 | 73.2 | 99 | 65.6 | 193 | 64.3 | 401 | 66.8 |
|  | Yes | 40 | 28.6 | 52 | 34.4 | 107 | 35.7 | 199 | 33.2 |
|  | **Implementation** |  |  |  |  |  |  |  |  |
|  | No | 74 | 49.7 | 95 | 62.9 | 136 | 45.3 | 305 | 50.8 |
|  | Yes | 75 | 50.3 | 56 | 37.1 | 164 | 54.7 | 295 | 49.2 |
|  | **Benefits** |  |  |  |  |  |  |  |  |
|  | No | 116 | 77.9 | 109 | 72.2 | 236 | 78.7 | 461 | 76.8 |
|  | Yes | 33 | 22.1 | 42 | 27.8 | 64 | 21.3 | 139 | 23.2 |
|  | **Evaluation** |  |  |  |  |  |  |  |  |
|  | No | 117 | 78.5 | 109 | 72.2 | 234 | 78 | 460 | 76.7 |
|  | Yes | 32 | 21.5 | 42 | 27.8 | 66 | 22.0 | 140 | 23.3 |
|  | **Involvement** |  |  |  |  |  |  |  |  |
|  | Not at all | 67 | 45.0 | 71 | 47.0 | 131 | 43.7 | 269 | 44.8 |
|  | Partly | 53 | 35.6 | 55 | 36.4 | 108 | 36.0 | 216 | 36.0 |
|  | All processes | 29 | 4.8 | 25 | 16.6 | 61 | 20.3 | 115 | 19.2 |

**Table 4** Clinical Characteristics of the Participants Categorized by Hospital Level (n=600)

| **Clinical Characteristics** | | | **Hospital Level** | | | | | | | | | | | **Total**  **(n=600)** | | |
| --- | --- | --- | --- | --- | --- | --- | --- | --- | --- | --- | --- | --- | --- | --- | --- | --- |
|  |  |  | **First-level**  **(n=149)** | | | | **Middle-level**  **(n=151)** | | | | **High- level**  **(n=300)** | | |  |  |  |
|  | | **Count** | | **%** | | **Count** | | **%** | | **Count** | | **%** | | **Count** | **%** | |
| **Comorbid burden** | | | | | | | | | | | | | | | | |
|  | no comorbid | | 1 | | 0.7 | | 0 | | 0.0 | | 8 | | 2.7 | 9 | | 1.5 |
|  | mild comorbid | | 141 | | 94.6 | | 139 | | 92.1 | | 234 | | 78.0 | 514 | | 85.7 |
|  | moderate comorbid | | 7 | | 4.7 | | 12 | | 7.9 | | 51 | | 17.0 | 70 | | 11.7 |
|  | high-comorbid | | 0 | | 0.0 | | 0 | | 0.0 | | 7 | | 2.3 | 7 | | 1.1 |
|  | **Chronic diseases** | |  | |  | |  | |  | |  | |  |  | |  |
|  | DM | | 14 | | 9.4 | | 19 | | 12.6 | | 25 | | 8.3 | 58 | | 9.7 |
|  | HT | | 40 | | 26.8 | | 12 | | 7.9 | | 15 | | 5.0 | 67 | | 11.2 |
|  | DLP | | 1 | | 0.7 | | 0 | | 0.0 | | 3 | | 1.0 | 4 | | 0.7 |
|  | DM & HT | | 17 | | 11.4 | | 63 | | 41.7 | | 59 | | 19.7 | 139 | | 23.2 |
|  | DM & DLP | | 8 | | 5.4 | | 8 | | 5.3 | | 28 | | 9.3 | 44 | | 7.3 |
|  | HT & DLP | | 46 | | 30.9 | | 2 | | 1.3 | | 35 | | 11.7 | 83 | | 13.8 |
|  | DM & HT & DLP | | 23 | | 15.4 | | 47 | | 31.1 | | 135 | | 45.0 | 205 | | 34.2 |

**Table 5** Polypharmacy of the Participants Categorized by Hospital Level (n=600)

| **Polypharmacy** | | **Hospital level** | | | | | | **Total**  **(n=600)** | |
| --- | --- | --- | --- | --- | --- | --- | --- | --- | --- |
|  |  | **First-level**  **(n=149)** | | **Middle-level**  **(n=151)** | | **High- level**  **(n=300)** | |  |  |
|  | | **count** | **%** | **count** | **%** | **count** | **%** | **count** | **%** |
|  | < 5 drugs | 70 | 47.0 | 56 | 37.1 | 78 | 26.0 | 204 | 34.0 |
|  | ≥ 5 drugs | 79 | 53.0 | 95 | 62.9 | 222 | 74.0 | 369 | 66.0 |

**Table 6** Dietary Patterns of the Participants Categorized by Hospital Level (n=600)

| **Dietary patterns** | **Hospital Level** | | | | | | **Total**  **(n=600)** | |
| --- | --- | --- | --- | --- | --- | --- | --- | --- |
|  | **First-level**  **(n=149)** | | **Middle-level**  **(n=151)** | | **High- level**  **(n=300)** | |  |  |
|  | **Count** | **%** | **Count** | **%** | **Count** | **%** | **Count** | **%** |
| No Mediterranean style | 83 | 55.7 | 94 | 62.3 | 151 | 50.3 | 328 | 54.7 |
| Mediterranean style | 66 | 44.3 | 57 | 37.7 | 149 | 49.7 | 272 | 45.3 |

**Table 7** Physical Activity of the Participants Categorized by Hospital Level (n=600)

| **Physical activity** | **Hospital level** | | | | | | **Total**  **(n=600)** | |
| --- | --- | --- | --- | --- | --- | --- | --- | --- |
|  | **First-level**  **(n=149)** | | **Middle-level**  **(n=151)** | | **High- level**  **(n=300)** | |  |  |
|  | **count** | **%** | **count** | **%** | **count** | **%** | **count** | **%** |
| Non-exercise | 78 | 52.3 | 75 | 49.7 | 185 | 61.7 | 338 | 56.3 |
| Exercise | 71 | 47.7 | 76 | 50.3 | 115 | 38.7 | 262 | 43.7 |

**Table 8** Medication Adherence of the Participants Categorized by Hospital Level (n=600)

| **Medication Adherence**  **(times/month)** | | **Hospital level** | | | | | | **Total**  **(n=600)** | |
| --- | --- | --- | --- | --- | --- | --- | --- | --- | --- |
|  |  | **First-level**  **(n=149)** | | **Middle-level**  **(n=151)** | | **High- level**  **(n=300)** | |  |  |
|  |  | **Count** | **%** | **Count** | **%** | **Count** | **%** | **Count** | **%** |
| **Forget to take medicine** | | | | |  |  |  |  |  |
| never | | 100 | 67.1 | 116 | 76.8 | 195 | 65 | 411 | 68.5 |
| 1-2 | | 36 | 24.2 | 19 | 12.6 | 76 | 25.3 | 131 | 21.8 |
| 3-5 | | 9 | 6.0 | 8 | 5.3 | 22 | 7.3 | 39 | 6.5 |
| 6-9 | | 2 | 1.3 | 2 | 1.3 | 0 | 0.0 | 4 | 0.7 |
| 10-15 | | 1 | 0.7 | 2 | 1.3 | 3 | 1.0 | 6 | 1.0 |
| > 15 | | 1 | 0.7 | 4 | 2.6 | 4 | 1.3 | 9 | 1.5 |
| **Changing the dose on own needs** | | | |  |  |  |  |  |  |
|  | never | 140 | 94.0 | 146 | 96.7 | 289 | 96.3 | 575 | 95.8 |
|  | 1-2 | 6 | 4.0 | 0 | 0.0 | 7 | 2.3 | 13 | 2.2 |
|  | 3-5 | 0 | 0.0 | 1 | 0.7 | 2 | 0.7 | 3 | 0.5 |
|  | 6-9 | 0 | 0.0 | 0 | 0.0 | 0 | 0.0 | 0 | 0.0 |
|  | 10-15 | 0 | 0.0 | 0 | 0.0 | 1 | 0.3 | 1 | 0.2 |
|  | > 15 | 3 | 2.0 | 4 | 2.6 | 1 | 0.3 | 8 | 1.3 |
| **Stop taking medication** | | | |  |  |  |  |  |  |
|  | never | 138 | 92.6 | 140 | 92.7 | 288 | 96.0 | 566 | 94.3 |
|  | 1-2 | 4 | 2.7 | 2 | 1.3 | 5 | 1.7 | 11 | 1.8 |
|  | 3-5 | 2 | 1.3 | 3 | 2.0 | 4 | 1.3 | 9 | 1.5 |
|  | 6-9 | 0 | 0.0 | 1 | 0.7 | 0 | 0.0 | 1 | 0.2 |
|  | 10-15 | 1 | 0.7 | 0 | 0.0 | 1 | 0.3 | 2 | 0.3 |
|  | > 15 | 4 | 2.7 | 5 | 3.3 | 2 | 0.7 | 11 | 1.8 |
| **Not taking medicine on time** | | | |  |  |  |  |  |  |
|  | never | 119 | 79.9 | 127 | 84.1 | 179 | 59.7 | 425 | 70.8 |
|  | 1-2 | 16 | 10.7 | 12 | 7.9 | 84 | 28.0 | 112 | 18.7 |
|  | 3-5 | 8 | 5.4 | 5 | 3.3 | 30 | 10.0 | 43 | 7.2 |
|  | 6-9 | 2 | 1.3 | 0 | 0.0 | 2 | 0.7 | 4 | 0.7 |
|  | 10-15 | 2 | 1.3 | 2 | 1.3 | 3 | 1.0 | 7 | 1.2 |
|  | > 15 | 2 | 1.3 | 5 | 3.3 | 2 | 0.7 | 9 | 1.5 |
| **Not taking all kinds of medicines** | | |  |  |  |  |  |  |  |
|  | never | 115 | 77.2 | 123 | 81.5 | 238 | 79.3 | 476 | 79.3 |
|  | 1-2 | 22 | 14.8 | 11 | 7.3 | 37 | 12.3 | 70 | 11.7 |
|  | 3-5 | 6 | 4.0 | 8 | 5.3 | 17 | 5.7 | 31 | 5.2 |
|  | 6-9 | 0 | 0.0 | 1 | 0.7 | 0 | 0.0 | 1 | 0.2 |
|  | 10-15 | 2 | 1.3 | 2 | 1.3 | 3 | 1.0 | 7 | 1.2 |
|  | > 15 | 4 | 2.7 | 6 | 4.0 | 5 | 1.7 | 15 | 2.5 |
| **Not taking medicines every meal** | | | |  |  |  |  |  |  |
|  | never | 105 | 70.5 | 117 | 77.5 | 178 | 59.3 | 400 | 66.7 |
|  | 1-2 | 29 | 19.5 | 17 | 11.3 | 91 | 30.3 | 137 | 22.8 |
|  | 3-5 | 7 | 4.7 | 8 | 5.3 | 22 | 7.3 | 37 | 6.2 |
|  | 6-9 | 3 | 2.0 | 1 | 0.7 | 1 | 0.3 | 5 | 0.8 |
|  | 10-15 | 3 | 2.0 | 2 | 1.3 | 37.5 | 1.0 | 8 | 1.3 |
|  | > 15 | 2 | 1.3 | 6 | 4.0 | 5 | 1.7 | 13 | 2.2 |
| **Not come for doctor visits** | | | |  |  |  |  |  |  |
|  | never | 141 | 94.6 | 149 | 98.7 | 244 | 81.3 | 534 | 89.0 |
|  | very few | 8 | 5.4 | 1 | 0.7 | 51 | 17.0 | 60 | 10.0 |
|  | few | 0 | 0.0 | 0 | 0.0 | 3 | 1.0 | 3 | 0.5 |
|  | some | 0 | 0.0 | 0 | 0.0 | 1 | 0.3 | 1 | 0.2 |
|  | often | 0 | 0.0 | 1 | 0.7 | 1 | 0.3 | 2 | 0.3 |
|  | very often | 0 | 0.0 | 0 | 0.0 | 0 | 0.0 | 0 | 0.0 |
| **No medicine and not taking it due to not coming for doctor visits** | | | | | | |  |  |  |
|  | never | 144 | 96.6 | 150 | 99.3 | 265 | 88.3 | 559 | 93.2 |
|  | very few | 4 | 2.7 | 0 | 0.0 | 28 | 9.3 | 32 | 5.3 |
|  | few | 0 | 0.0 | 0 | 0.0 | 4 | 1.3 | 4 | 0.7 |
|  | some | 1 | 0.7 | 0 | 0.0 | 1 | 0.3 | 2 | 0.3 |
|  | often | 0 | 0.0 | 1 | 0.7 | 1 | 0.3 | 2 | 0.3 |
|  | very often | 0 | 0.0 | 0 | 0.0 | 1 | 0.3 | 1 | 0.2 |
| **Medication adherence** | | | |  |  |  |  |  |  |
|  | No | 17 | 11.4 | 19 | 12.6 | 45 | 15.0 | 81 | 13.5 |
|  | Yes | 132 | 88.6 | 132 | 87.4 | 225 | 85.0 | 519 | 86.5 |
